# Retinoic acid associates with mortality of patients on long-term hemodialysis

**DOI:** 10.1080/0886022X.2022.2126786

**Published:** 2022-11-04

**Authors:** Marta Kalousová, Miroslava Zelenková, Aleš A. Kuběna, Sylvie Dusilová-Sulková, Vladimír Tesař, Tomáš Zima

**Affiliations:** a Institute of Medical Biochemistry and Laboratory Diagnostics, First Faculty of Medicine, Charles University and General University Hospital in Prague, Prague, Czech Republic; b Department of Nephrology, University Hospital Hradec Králové and Charles University, Faculty of Medicine in Hradec Králové, Hradec Králové, Czech Republic; c Department of Nephrology, First Faculty of Medicine, Charles University and General University Hospital in Prague, Prague, Czech Republic

**Keywords:** ATRA, cardiovascular, hemodialysis, mortality, outcome, retinol, retinoic acid

## Abstract

**Background:**

Retinol concentrations in serum are significantly higher in patients on hemodialysis (HD) compared to healthy controls. Its lower concentrations have been reported to be an independent predictor of mortality. ATRA – all-trans retinoic acid – is an important compound related to retinol. The objective was to determine ATRA concentrations in serum and to find their association with the prognosis of patients on long-term HD.

**Methods:**

ATRA was determined by high-performance liquid chromatography in a group of 247 HD patients (follow-up five years) and 54 healthy controls.

**Results:**

Although serum retinol concentrations were higher in the studied cohort of HD patients, ATRA was lower – median 1.13 (interquartile range 0.90–1.60) ng/mL in HD patients versus 1.42 (1.08–1.63) ng/mL in healthy controls, *p* = 0.02. Lower ATRA was significantly related to overall mortality of HD patients (HR (95%CI) 0.63 (0.47–0.85) per interquartile range, *p* = 0.003). The best prognosis was observed in patients with concentrations of both ATRA and retinol above the median (*p* = 0.003).

**Conclusions:**

We detected decreased retinoic acid levels in HD patients compared to healthy controls. Lower concentrations of ATRA represent a significant predictor of mortality and provide additional information to retinol.

## Introduction

The population of patients with chronic kidney diseases is known for its higher morbidity and mortality, which is related to the degree of decline in kidney function, and is the highest in patients with end-stage renal disease. Many biomarkers have been tested to demonstrate their relationship to kidney function and their significance in these patients. Retinol represents one of these important biomarkers. Its serum concentrations are significantly higher in patients on long-term hemodialysis (HD) compared to healthy controls and its lower concentrations have been reported to be an independent predictor of mortality [1,2].

Retinol (vitamin A) is a fat-soluble vitamin with several functions in the organism. It is involved in the visual process, in cell growth and differentiation, and belongs to important antioxidants. It is stored in the liver. In plasma, it is transported by the retinol-binding protein (RBP). Retinol serves as a precursor for retinal, which is part of the eye pigment rhodopsin and retinoic acid (all-trans retinoic acid, ATRA being the most import one), which is biologically active.

ATRA is formed from retinol in a two-step oxidation: in the first, retinol is oxidized by alcohol dehydrogenase to retinal (retinaldehyde) and in the second catalyzed by retinal dehydrogenase, retinoic acid rises. ATRA serves as a lipophilic hormone, binding to the intracellular retinoic acid receptor (RAR), and transcriptionally regulating cell proliferation, differentiation, and immune response. ATRA and its isomers have been used for many years for the treatment of psoriasis, acne, acute promyelocytic leukemia, as well as some solid tumors [3,4]. Despite beneficial effects and general good tolerability, in some cases, the treatment may be accompanied by a complication known as retinoic acid syndrome (RAS) which also includes acute renal failure [5,6]. This syndrome was described in both adults [5] and children (a case with signs of nephrotic range proteinuria) [7]. On the other hand, tissue culture and animal studies show the protective effect of ATRA on the progression of kidney damage [8]. Additionally, the role of ATRA in the reduction of atherosclerotic plaques [9] and its dose-dependent effect on cardiac remodeling [10] was also demonstrated. As data on ATRA levels in patients with renal diseases are limited [11] and cardiovascular disease is the main reason for high mortality of patients with advanced renal diseases, our aim was to measure ATRA concentrations in serum from long-term HD patients and to determine their prognostic significance.

## Methods

### Design of the study and participants

This study follows on from our previous study that demonstrated the negative impact of low retinol on the prognosis of HD patients [1]. Patients from six dialysis centers in the Czech Republic entered the prospective observational cohort study in November/December 2003. The sample size was calculated for the area of interest of the hazard ratio 1.5–2, for a single parameter hypothesis, and for power 80% and 90% [12] assuming 50% mortality of HD patients during 5 years. Blood samples and clinical and laboratory data from patient records were collected at the beginning of the study and the study lasted 5 years until November/December 2008. A detailed description of the study including the CONSORT flow diagram was previously published [1]. Due to the date of the study and the previous use of samples, not all samples were available, leading to a slightly decreased number of subjects. For this reason, finally 247 HD patients (117 women and 130 men) with a median age of 65 (54–74) years and 54 healthy subjects (36 women and 18 men) with a median age of 59 (53–63) years were included in the present study.

The causes of renal failure in HD patients were 25% interstitial nephritis, 20% glomerulonephritis, 19% diabetic nephropathy, 15% polycystic kidney disease, 9% hypertensive nephropathy, and in other patients combined. Median residual diuresis was 0.5 l per 24 h. The patients were on dialysis from 1 month to 27 years, a median 2 years and almost 90% of them were dialyzed *via* a native arteriovenous fistula. Their median Kt/V was 1.29. Almost all patients (88%) were dialyzed with low-flow dialyzers. In 46% of the cases, polysulfone membranes were used, in 33% of the cases, diacetate cellulose membranes, and in other cases triacetate cellulose, polyamide, or polymethylmetacrylate dialyzers were used. Regarding the basic clinical characteristics of HD patients, briefly, 84% of patients had hypertension, one third of the patients were diabetics, 39% had dyslipidemia, and 11% were malnourished. 61% of the patients had cardiovascular disease in their history, 25% had cerebrovascular disease, and 26% had peripheral vascular disease. 21% of the patients were smokers. HD patients received the usual drug treatment which changed over time as appropriate. Statins were taken by about a quarter of the patients and ACE inhibitor or ARII blockers by approximately 51% of the patients. They did not receive any vitamin A supplementation and no retinoic acid was administered. The comparison of basic characteristics and laboratory parameters between patients and controls is shown in Table 1. Follow-up data revealed that 141 patients (i.e. 57%) died during the 5-year study, and in half of them (70 patients) the cause of death was cardiovascular. Other causes of death were infection (39 patients), cancer (14 patients), or other. During follow-up, 48 patients received kidney transplantation and 7 of them died (in 5 of them the cause was infection, in 1 cardiovascular event and in 1 tumor). One patient was censored from the study due to withdrawal from dialysis.

**Table 1.** Basic characteristics and results of laboratory examinations of the studied groups.

	HD patients	Controls	*p*
Number of subjects (men/women)	247 (130/117)	54 (18/36)	
Age (years)	65 (54–74)	59 (53–63)	0.018
BMI (kg/m^2^)	24.6 (22.1–27.9)	25.3 (23.6–28.3)	0.264, n.s.
Leukocytes (x10^9^/L)	6.7 (5.5–7.9)	6.0 (5.0–7.9)	0.044
Hemoglobin (g/L)	105 (96–114)	138 (134–144)	*p* < 0.001
Hematocrite	0.31 (0.29–0.34)	0.41 (0.39–0.43)	*p* < 0.001
Thrombocytes (x10^9^/L)	196 (161–249)	251 (222–277)	*p* < 0.001
Creatinine (µmol/L)	752 (624–875)	86 (77–98)	*p* < 0.001
Urea (mmol/L)	22.3 (17.8–26.5)	5.0 (4.2–5.6)	*p* < 0.001
Uric acid (µmol/L)	385 (325–429)	255 (231–318)	*p* < 0.001
Albumin (g/L)	37.8 (35.5–40.0)	44.0 (43.0–46.1)	*p* < 0.001
Transferrin (g/L)	1.75 (1.55–1.98)	2.72 (2.48–3.04)	*p* < 0.001
Calcium (mmol/L)	2.31 (2.18–2.45)	2.35 (2.31–2.43)	0.055, n.s.
Glucose (mmol(L)	5.5 (4.8–6.8)	4.9 (4.7–5.3)	0.302, n.s.
Cholesterol (mmol/L)	4.6 (3.9–5.3)	5.6 (5.1–6.3)	*p* < 0.001
LDL-cholesterol (mmol/L)	2.5 (1.9–3.1)	3.2 (2.8–3.8)	*p* < 0.001
HDL-cholesterol (mmol/L)	1.2 (1.1–1.5)	1.7 (1.5–1.9)	*p* < 0.001
Triacylglycerols (mmol/L)	1.77 (1.27–2.32)	1.09 (0.88–1.60)	*p* < 0.001
Bilirubin (µmol/L)	9.3 (8.0–11.0)	10.3 (8.5–13.1)	0.010
ALT (µkat/L)	0.29 (0.21–0.40)	0.37 (0.28–0.45)	0.001
CRP (mg/L)	4.4 (2.6–9.7)	2.4 (1.9–3.7)	*p* < 0.001
Fibrinogen (g/L)	4.2 (3.3–5.3)	3.4 (3.0–3.9)	*p* < 0.001
Orosomucoid (g/L)	1.09 (0.89–1.33)	0.79 (0.66–0.93)	*p* < 0.001
Retinol (mg/L)	1.6 (1.2–2.1)	1.0 (0.8–1.2)	*p* < 0.001
RBP4 (mg/L)	119.2 (105.7–132.6)	42.3 (37.1–48.0)	*p* < 0.001

Results are expressed as median (IQR). Comparison between groups was done with the Mann–Whitney test. Reference ranges of routine laboratory parameters in the laboratory in which the analysis was performed: leukocytes (×10^9^/L) M 4.0–10.2, F 4.0–10.7; hemoglobin (g/L) M 135–174, F 116–163; hematocrite M 0.39–0.51, F 0.33–0.47; thrombocytes (×10^9^/L) M 142–327, F 131–364; creatinine (µmol/L) M 44–110, F 44–104; urea (mmol/L) M 2.8–8.0, F 2.0–6.7; uric acid (µmol/L) M 220–420, F 140–340; albumin (g/L) 35.0–53.0; transferrin (g/L) 2.0–3.6; calcium (mmol/l) 2.0–2.75; Glucose (mmol/L) 3.9–5.6; cholesterol (mmol/L) 3.8–5.8; LDL-cholesterol (mmol/L) M 2.2–4.3; F 2.2–4.5; HDL-cholesterol (mmol/L) M 1.1–2.1, F 1.3–2.3; triacylglycerols (mmol/L) 0.68–1.69; bilirubin (µmol/L) 2.0–17.0; ALT (µkat/L) 0.10–0.78; CRP (mg/L) 0.0–7.0 (routine method); fibrinogen (g/L) 2.0–4.0; orosomucoid (g/L) 0.5–1.2; retinol (mg/L) 0.4–1.2. ALT: alanine aminotransferase; AST: aspartate aminotransferase; BMI: body mass index; CRP: C-reactive protein; F: female; HD: hemodialysis; HDL: high-density lipoprotein; IQR: interquartile range; LDL: low-density lipoprotein; M: male; n.s.: not significant; RBP4: retinol binding protein 4.

### Statement of ethics

The study adheres to the principles of the Declaration of Helsinki and was carried out with the agreement of the Ethics Committee of the General University Hospital in Prague (No. 26/03 and No. 352/11 S-IV). All subjects gave their informed written consent to participate in the study.

### Laboratory analyses

Blood was collected from HD patients at the beginning of the study. Samples were taken at the same time as blood samples for routine laboratory examinations prior to the dialysis session and before heparin administration. The patients were asked not to eat before, but during the dialysis session. In the control group, standard blood collection was performed in the morning after fasting overnight. The serum aliquots were stored frozen at −80 °C until they were used for the measurement of ATRA.

Due to the long storage of the samples (blood collection 2003 and analysis 2017), fresh sera from 45 HD patients and 17 healthy subjects (rest material from basic biochemical examinations) were used for ATRA measurement to be informed of whether and how much the storage could influence the result.

Retinoic acid (ATRA) was determined according to Zelenková et al. as previously described [13]. Briefly, ATRA was measured in serum after deproteination with acetonitrile followed by extraction with hexane and ethylacetate (Sigma, St. Louis, MO, www.sigma.aldrich.com) by high performance liquid chromatography (HPLC) on a Hewlett Packard apparatus (Waldbronn, Germany). ATRA was separated in an Agilent Eclipse XDB-C_18_/5 µm 4.6*150 mm column at 37 °C and detected with the diode detector DAD at 355 nm. This method has a detection limit of 0.21 ng/ml, a quantification limit of 0.70 ng/ml and linearity for ATRA concentration 0.78–25 ng/ml. The coefficient of variation within the run was 4.31% and between the runs 9.55%.

The determination of other parameters was previously described [1]. Briefly, retinol was measured by high performance liquid chromatography (HPLC), retinol binding protein 4 by enzyme-linked immunosorbent assay (ELISA), and C-reactive protein turbidimetrically on latex particles. Routine biochemical and hematological parameters were measured using standard laboratory methods on automated analyzers.

### Statistics

Statistical software Wolfram Mathematica 11.3 (UK) was used for statistical analysis. For several samples, the ATRA concentration was below the limit of detection. We included these samples in the analyzes to avoid bias and adapted statistical methods to work with non-detect data [14]. For this reason, and also because of the statistical distributions far from normal, we preferred nonparametric methods.

The aggregate characteristics of the numerical variables are presented in the form of median (lower, upper quartile). Differences in concentrations between groups were tested with the Mann-Whitney test and by the Kruskal–Wallis test for comparison of more subgroups. The degree of correlation between two numerical variables was evaluated using Kendall’s τ and corresponding test.

Survival and cause-specific mortality were analyzed using the Kaplan–Meyer analysis and Cox regression. Comparison of the survival of subgroups of patients below and above the median concentration or between quartiles of concentrations was performed by the Kaplan–Mayer analysis. The log-rank test was used tocompare survival curves. The *post hoc* power was calculated as well. In the Cox regression, the concentrations of molecules as an independent predictor were transformed to the value of the cumulative distribution function of their concentrations. The Cox regression estimation of the relative risk ratio is present in the form of HR (hazard ratio) and a 95% confidence interval per interquartile range. *p* < 0.05 was standardly taken as the limit for the statistical significance of the results.

## Results

**Figure 1.**
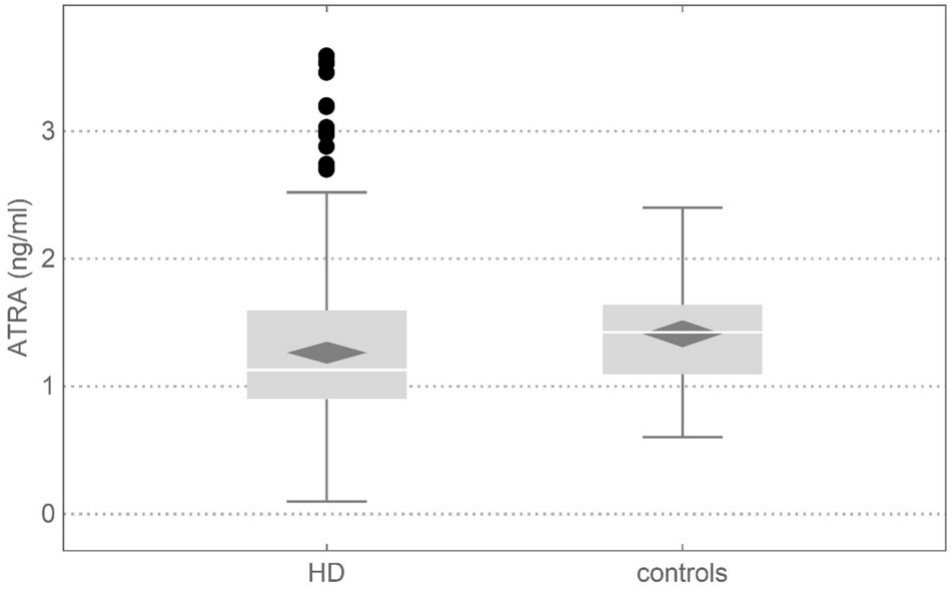
ATRA in HD patients and healthy subjects. HD 1.13 (0.90–1.60) ng/mL versus controls 1.42 (1.08–1.63) ng/mL, *p* = 0.02. ATRA: all-trans retinoic acid; HD: hemodialysis.

Although serum retinol concentrations were higher in the studied cohort of HD patients, ATRA was lower – median 1.13 (interquartile range 0.90–1.60) ng/mL in HD patients versus 1.42 (1.08–1.63) ng/mL in healthy controls, *p* = 0.02), Figure 1. In HD patients, ATRA correlated significantly both with retinol (*τ* = 0.245, *p* < 0.001) and with RBP4 (*τ* = 0.258, *p* < 0.001), while both relationships in controls were not statistically significant.

The ATRA concentrations did not differ between men and women. Patients with cardiovascular disease in their personal history had significantly lower ATRA levels (*p* = 0.015), but no effect of ACE inhibitors was observed. Dyslipidemia significantly increased ATRA levels (*p* = 0.007), but statin treatment did not influence it. We did not show any difference between patients with and without diabetes mellitus and with and without hypertension, and there was no relationship of ATRA concentrations with the causes of renal failure. Malnutrition did not have an impact on ATRA levels (Table 2). There was no correlation of ATRA with the duration of the dialysis treatment.

**Table 2.** ATRA (ng/mL) in subgroups of HD patients.

	Yes	No	*p*
Men/women	Men 1.21 (0.94–1.71)		0.110, n.s.
	Women 1.08 (0.83–1.51)		
Cardiovascular disease in patients’ history	1.07 (0.79–1.53)	1.23 (0.96–1.73)	0.015
ACE inhibitors	1.20 (0.93–1.64)	1.09 (0.84–1.51)	0.253, n.s.
Dyslipidemia	1.26 (1.00–1.71)	1.08 (0.80–1.50)	0.007
Statin treatment	1.25 (0.94–1.54)	1.12 (0.86–1.66)	0.408, n.s.
Diabetes mellitus	1.10 (0.84–1.61)	1.18 (0.91–1.55)	0.615, n.s.
Hypertension	1.15 (0.90–1.59)	1.08 (0.90–1.63)	0.840, n.s.
Malnutrition	1.04 (0.77–1.70)	1.16 (0.91–1.59)	0.440, n.s.
Causes of renal failure	Interstitial nephritis 1.09 (0.76–1.54)		0.223, n.s.
	Glomerulonephritis 1.26 (0.99–1.59)		
	Diabetic nephropathy 1.14 (0.82–1.72)		
	Polycystic kidney disease 1.05 (0.84–1.51)		
	Hypertensive nephropathy 1.35 (1.09–1.73)		
	Combined causes 0.96 (0.79–1.42)		

Results are expressed as median (IQR). Comparison between groups was done with Mann–Whitney test and Kruskal–Wallis test in case of more subgroups. ATRA: all-trans retinoic acid; HD: hemodialysis; n.s.: not significant.

**Figure 2.**
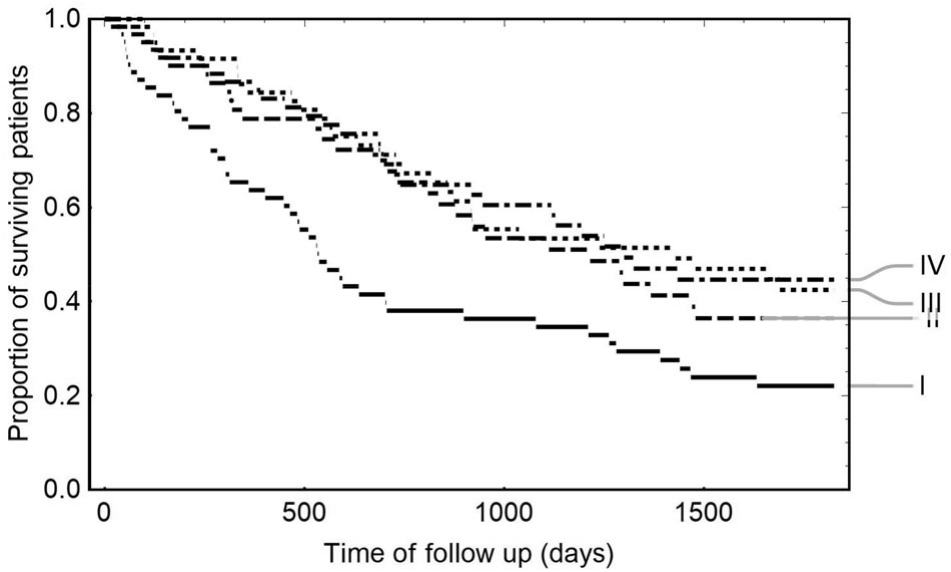
Kaplan–Meier analysis of the significance of ATRA in the survival of hemodialysis patients. Survival curves of HD patients are stratified by quartiles of ATRA concentration: I … ATRA ≤ 0.9 ng/mL; II … 0.9 ng/mL < ATRA ≤ 1.13 ng/mL; III … 1.13 ng/mL < ATRA ≤1.595 ng/mL; IV … 1.595 ng/mL < ATRA; *p* = 0.002 (log-rank test); ATRA: all-trans retinoic acid; HD: hemodialysis.

Low ATRA concentrations worsened the survival of HD patients. Kaplan-Meier analysis revealed that patients with ATRA concentrations above median (ATRA > 1.13 ng/mL) survived significantly longer than those with ATRA below median (1323 versus 808 days, *p* = 0.006) (HR 1.604, power 78%). Additionally, a finer division into subgroups by ATRA concentration quartiles maintains significance (*p* = 0.002) and shows that the critical subgroup is the first quartile (ATRA ≤ 0.9 ng/mL), Figure 2. The revised cutoff (the lowest quartile compared to three upper quartiles) even more impacts survival prediction: the median survival is now 536 days (first ATRA quartile) versus 1288 days (other ATRA quartiles), *p* < 0.001. Further analysis showed that the lowest ATRA quartile was also the worst with respect to cardiovascular mortality (*p* = 0.014). However, mortality from infection was not affected by ATRA concentrations (*p* = 0.24).

**Figure 3.**
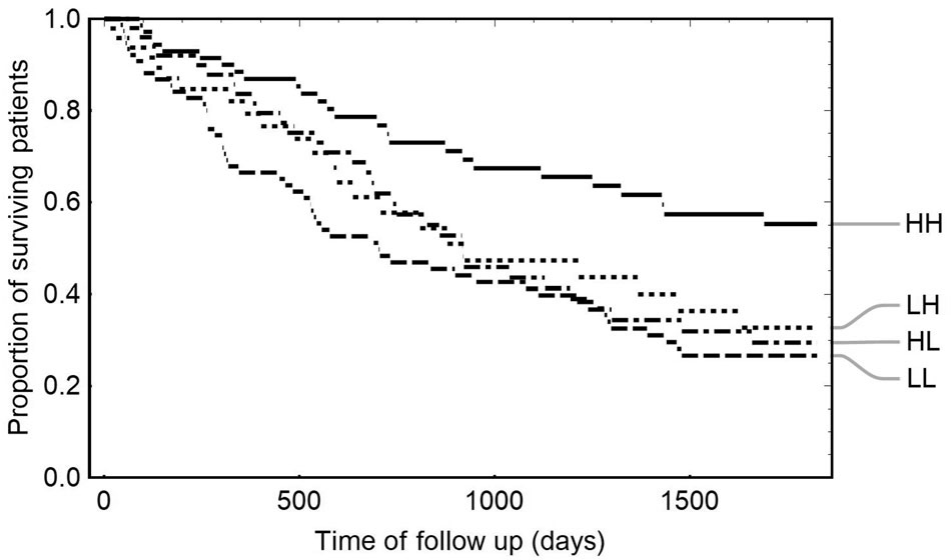
Kaplan–Meier analysis of the significance of ATRA and retinol in the survival of hemodialysis patients. Survival curves of HD patients are stratified by medians of ATRA (1.13 ng/mL) and retinol (1.6 mg/L) concentrations. HH (high ATRA, high retinol): ATRA >1.13 ng/mL, retinol >1.6 mg/L; HL (high ATRA, low retinol): ATRA >1.13 ng/mL, retinol ≤1.6 mg/L; LH (low ATRA, high retinol): ATRA ≤1.13 ng/mL, retinol >1.6 mg/L; LL (low ATRA, low retinol): ATRA ≤1.13 ng/mL, retinol ≤1.6 mg/L; *p* = 0.003 (log-rank test); ATRA: all-trans retinoic acid; HD: hemodialysis.

In further analysis, ATRA was evaluated together with retinol. There is a significant difference in survival between 2 × 2 groups defined by the combination of ATRA median (1.13 ng/mL) and retinol median (1.6 mg/L) cuts (*p* = 0.003), Figure 3. The group of HD patients with high levels of ATRA and high levels of retinol has the best prognosis: the majority of the group outlives the study time, that is, the median survival of the high-high group exceeds 1825 days, while the median survival of the remaining group is 825 days (*p* < 0.001).

The Cox regression shows a lower ATRA as a significant negative prognostic factor for overall mortality of HD patients (HR (95%CI) 0.63 (0.47–0.85) per interquartile range, *p* = 0.003), i.e. the death risk decreases with increasing ATRA concentration, namely 1.59 times per interquartile range (95% CI 1.18, 2.14). After including retinol in the model, the estimated hazard ratio drops to 1.32, 95% CI 0.96–1.82, and the impact of ATRA becomes insignificant (*p* = 0.089). The current model shows retinol as a protective factor; higher concentration of retinol decreases the risk of death 1.77 times per interquartile range, 95% CI 1.26–2.50, *p* = 0.001.

*Technical note*: in fresh samples from HD patients, ATRA was higher compared to the study samples from HD patients 1.62 (1.36–2.19) ng/mL (*p* < 0.001), however, the concentrations in fresh control samples were comparable 1.57 (1.35–1.76) ng/mL with the study control samples (*p* = 0.13, not significant).

## Discussion

This is the first study to demonstrate the prognostic significance of lower ATRA levels in patients on long-term HD. The best prognosis was observed in patients with high ATRA and high retinol concentrations (above median values). This fact points to the importance of both molecules. Data on ATRA levels in patients with kidney diseases are limited and studies dealing with ATRA in patients not treated with ATRA are also rare. Although ATRA might be of interest, its measurement is demanding as no commercial kit is available for its determination, and only methods such as high-performance liquid chromatography (HPLC) or mass spectrometry can be used. The measurement presented in this paper was performed using a new HPLC method developed in our laboratory [13].

To our knowledge, there is only one study in which ATRA was evaluated in patients with chronic kidney disease, including a small group of 10 HD patients and 21 healthy controls [11]. Measurement was carried out with LC-MS/MS and ATRA concentrations in both HD patients and controls were lower than in our current report (median 3.0 nM, that is, 0.9 ng/mL for HD patients and 1.7 nM, that is, 0.51 ng/mL for controls). However, other studies report rather higher serum ATRA values, for example 2.0 ng/ml in patients with acute ischemic stroke [15], 3.5 ng/ml in 37 healthy volunteers [16], or 4.9 ng/mL in 12 healthy volunteers [17], suggesting the need for the development and use of standard reference material in future clinical studies to ensure reproducibility of the results.

Our results are in line with the general protective effect of elevated serum ATRA levels against chronic conditions associated with inflammation and oxidative stress. In a recent study, Hou et al. demonstrated the link between low ATRA concentrations in serum and post-stroke cognitive impairment in ischemic stroke patients [15]. Furthermore, low serum levels of ATRA were associated with an increased risk of overall mortality and mortality from cardiovascular causes in patients with acute ischemic stroke [18]. Although a relationship between the renin-angiotensin-aldosterone system and ATRA has been described in rats [19], in our study no effect of treatment with ACE inhibitors was shown. In addition, ATRA has been described to reduce atheroma plaques in an animal model of atherosclerosis [9]. We have shown a significant relationship between ATRA and dyslipidemia, but we did not observe differences in ATRA levels in statin-treated patients and those without statin treatment. A combination of high ATRA and high retinol concentrations is the most beneficial for the prognosis of patients. Low ATRA and high retinol are as bad as high ATRA and low retinol or low levels of both molecules. Lower ATRA levels despite higher retinol levels in HD patients could be due to metabolic changes in the uremic environment that might lead to a decrease in the formation of this important biologically active substance. However, ATRA as a special parameter provides additional prognostic information to retinol, which can be routinely measured in laboratories of clinical biochemistry.

We must mention that the present study has several limitations. Our aim was to measure ATRA in the same group where retinol was previously measured to enable the comparison of both parameters [1]. However, not all samples were available, leading to about 5% decrease of HD patients (261 versus 247 patients) and also slight decrease of controls, which we think is acceptable. The samples in controls were collected after fasting overnight, which was not always fulfilled in HD patients (afternoon and night sessions); however, patients were asked to eat not before, but during the dialysis session and there was no significant difference in serum glucose suggesting also limited effect on other parameters. Another drawback is that the samples were stored for almost 14 years (collection in 2003 and analysis in 2017) at −80 °C and there are no data whether and how much the storage could have influenced the results. For this reason, we also have analyzed fresh samples, sera remaining from routine biochemical examinations. The levels in healthy controls were comparable (slightly higher), but the levels in fresh sera from HD patients were markedly higher than in the study samples, which, based on our results, would indicate a better prognosis. The serums of both HD patients and controls were processed and stored in the same way, so if it was a storage problem, both groups should be influenced, which was not the case. Factors affecting ATRA in the organism are not well known, but we assume an improvement in the treatment of HD patients over years (drugs as well as dialysis characteristics) and based on this also a better prognosis of current HD patients. We should mention mainly the following points: First, the change of hemodialysis with low flux membranes to hemodiafiltration using high-flux membranes, which because of convection, also allows removal of middle molecules. Second, reverse osmosis and ultrapure water was not available in the past, and water was refined just by demineralization which did not provide absolute safety regarding the presence of endotoxin or its fragments leading to subsequent induction of microinflammation. Third, less biocompatible membranes were used, which also increased the inflammatory process. Additionally, there were less tools to control secondary hyperparathyroidism, mineral bone disease, and vascular calcifications.

Our result of the comparison of old and fresh sera is very important not only for this study but also for the evaluation of the results of other studies. Results of studies are often compared in meta-analyses, however, our results indicate that if the studies are from different time periods, they may not be comparable without reservation. Not only the type of population and geographical origin of the study, different eating habits or time of storage of the samples, but also changes in the treatment of the specific group of patients over years should be considered.

## Conclusion

In summary, we detected decreased levels of retinoic acid in HD patients compared to healthy controls. However, these concentrations were comparable to the only study available in 10 HD patients [11]. Lower concentrations of ATRA represent a significant predictor of mortality and provide additional information to retinol. We suggest that not long-term storage, but advances in patient treatment over years may have caused the difference in ATRA levels between old and fresh samples and may indicate a better prognosis of current HD patients. Replication of our study in another patient population would be appropriate to shed light on the current situation of HD patients.

## Data Availability

Data are available with the corresponding author on reasonable request.
